# Performance Evaluation of BD Phoenix NMIC-413 Antimicrobial Susceptibility Testing Panel for Imipenem, Meropenem, and Ertapenem Against Clinical Carbapenem-Resistant and Carbapenem-Susceptible *Enterobacterales*

**DOI:** 10.3389/fmed.2021.643194

**Published:** 2021-04-14

**Authors:** Jingjia Zhang, Peiyao Jia, Ying Zhu, Ge Zhang, Yingchun Xu, Qiwen Yang

**Affiliations:** ^1^Department of Clinical Laboratory, Peking Union Medical College Hospital, Peking Union Medical College, Chinese Academy of Medical Sciences, Beijing, China; ^2^Graduate School, Peking Union Medical College, Chinese Academy of Medical Sciences, Beijing, China

**Keywords:** BD Phoenix NMIC-413, CRE, broth microdilution, disk diffusion, evaluation

## Abstract

**Purpose:** The infection of carbapenem-resistant *Enterobacterales* (CRE) has become a major clinical and healthcare problem worldwide. The screening methods of CRE have been extensively developed but still need improving [e.g., tests with accurate and simple minimum inhibitory (MICs)]. In this study, the performance of the BD Phoenix NMIC-413 AST panel was evaluated against clinical CRE and carbapenem-susceptible *Enterobacterales* (CSE) in China. The panel was first evaluated in the Chinese clinical lab.

**Methods:** Antimicrobial susceptibility testing of 303 clinical *Enterobacterales* isolates were conducted by broth microdilution (BMD), Phoenix NMIC-413 AST panel, and disk diffusion method for imipenem, ertapenem, and meropenem. Considering BMD is a gold standard, essential agreement (EA), categorical agreement (CA), minor error (MIE), major error (ME), and very major error (VME) were determined according to CLSI guidelines. CA and EA > 90%, ME <3%, and VME <1.5% were considered as acceptable criteria. Polymerase chain reaction and sanger sequencing were performed to determine the β-lactamase genotypes of CRE isolates.

**Results:** Three hundred and three isolates included 195 CREs and 108 CSEs were enrolled according to the BMD-MIC values of three carbapenems. Tested CREs showing 100 *bla*_KPC−2_-positive organisms, 31 *bla*_IMP_-positive organisms, 28 *bla*_NDM_-positive organisms, 5 *bla*_VIM_-positive organisms, 2 both *bla*_IMP_ and *bla*_VIM_-positive organisms, 2 *bla*_OXA−48_-positive organisms, and 27 isolates without carbapenemase genes. For the Phoenix NMIC-413 method, CA and EA rates >93%, MIE rates <5%, ME rates <1.75%, and VME rates were 0%, across the three drugs. For the disk diffusion method, the CA rates for three drugs were all >93%, while the MIE and ME rates were all <5 and <3%, respectively. VME rate was 3.28% for imipenem, exceeded the cut-off value specified by CLSI M52, 0 and 0.56% for ertapenem and meropenem, separately.

**Conclusion:** Based on the genomic data, the detection of CRE and CSE was more reliable using the BD Phoenix NMIC-413 panel compared to the BMD and disk approaches. Therefore, our study supports the use of BD Phoenix NMIC-413 panel as a suitable alternative to BMD for the detection of carbapenem resistant isolates in a clinical setting.

## Introduction

Carbapenem-resistant *Enterobacterales* (CRE) is a major clinical and public health issue worldwide, which can cause infections associated with high mortality and have limited treatment options (1, 2). CREs are generally resistant to all β-lactams, including carbapenems such as imipenem, meropenem, ertapenem, doripenem (3), and other antibiotics such as cephalosporins, quinolones, and aminoglycosides, which further restrict the choice of antibiotic treatment. After the initial report of KPC-1 (*Klebsiella pneumoniae* carbapenemase-1) from a strain of *K. pneumoniae* discovered in North Carolina in 2001 (4), CRE has been widely reported in almost every state (1). In China, the incidence of carbapenem-resistant *Escherichia coli* and *K. pneumoniae* increased from 0 and 0.7% in 2004 to 1.0 and 13.4% in 2014 (5, 6).

The mechanism of Carbapenem resistance can be divided into two types: carbapenemase factor and carbapenemase-non-producing factor. Carbapenem resistance in *Enterobacterales* is mainly mediated by the horizontal transfer of genes encoding carbapenemases, although porin mutations or overexpression of efflux pumps can lead to carbapenem resistance, especially in combination with the hyperproduction of β-lactamase (7). Carbapenemases consisted of different molecular classes: A, B, and D of the Ambler classification (8). The clinically most important and frequent carbapenemases in *Enterobacterales* are class A (KPCs), class B metallo-β-lactamases (VIM, IMP, and NDM), and class D (OXA-48) subgroups and their variants (9–12).

Recently, different methods were developed to detect CRE, such as the disk diffusion method, Brilliance TM CRE Agar, chromID Carba, and molecular methods (13, 14). However, phenotypic antimicrobial susceptibility assay which can accurately determine minimum inhibitory concentrations (MICs) is still the key method to guide clinical medication quickly and precisely. Most of the products in the market used to measure the MICs were based on the broth microdilution (BMD) or improved BMD method, such as BioMérieux VITEK 2, Beckman Coulter MicroScan WalkAway, and BD Phoenix. BD Phoenix NMIC-413 panel is a new panel that has recently been marketed, and covered the main cephalosporins and carbapenems such as imipenem, meropenem, and ertapenem. However, its performance to detect carbapenem susceptibility was not well-evaluated yet. In this study, the performance of the BD Phoenix NMIC-413 panel were evaluated using carbapenem-resistant and carbapenem-susceptible clinical isolates in China.

## Materials and Methods

### Isolates

The Human Research Ethics Committee of our hospital approved the study protocols (Et. Number: S-K677). Three hundred and three clinical *Enterobacterales* isolates from Peking Union Medical College Hospital from 2010–2019 were evaluated in this study. The majority of the specimens were taken from sputum (74, 24.42%), blood (59, 19.47%), urine (58, 19.14%), bronchoalveolar lavage fluid (33, 10.89%), peritoneal fluid (29, 9.57%), gall bladder (19, 6.27%), abscess (12, 3.96%), wound (6, 1.98%), and others (13, 4.29%). Strains were isolated from surgery department (115, 37.95%), medicine department (96, 31.68%), ICU (65, 21.45%), emergency department (21, 6.93%), and pediatric department (6, 1.98%) (Supplementary Table 1). Isolates were identified using MALDI-TOF MS (Vitek MS, BioMérieux, France). All duplicate isolates (the same genus and species from the same patient) were excluded. Isolates were stored at −80°C in a cryotube with 20% (w/v) skimmed milk until subcultured on Blood Agar Plate (Oxoid, Basingstoke, United Kingdom). *Klebsiella pneumoniae* BAA 1705 (*bla*_KPC−2_), *Es. coli* ATCC 2452 (*bla*_NDM−1_), *K. pneumoniae* ATCC 700603, and *Es. coli* ATCC 25922 were used as quality control strains.

### BD Phoenix System

the BD Phoenix NMIC-413 panel (BD Catalog Number: 448442) was used to determined MICs of imipenem (range: 0.25–8 mg/L), meropenem (range: 0.125–8 mg/L), and ertapenem (range: 0.25–2 mg/L) according to the manufacturer's instructions. In short, the identification broth was regulated with bacterial colonies from Blood Agar Plates to 0.5 McFarland standard using a spectrophotometric device. Transferred 25 μL 0.5 McFarland identification broth suspension to the Phoenix Antibiotic susceptibility testing (AST) broth, which was supplemented with 50 μL of the Phoenix AST indicator for the organism growth detecting before added to the panels. The panels were loaded into the Phoenix device (M50). The results were analyzed using Epicenter data management software version 6.61A (BD Diagnostic Systems) after 16 h of incubation (15, 16).

### BMD Method

The susceptibility of strains to imipenem (range: 0.12–128 mg/L), meropenem (range: 0.12–128 mg/L), and ertapenem (range: 0.12–128 mg/L) were tested and analyzed. Three antimicrobial powders were obtained from National Institutes for Food and Drug Control (Beijing, China). A 0.5 McFarland standard suspension was prepared and used to inoculate the reference BMD panel according to CLSI M100 (17). Incubated these panels at 35°C for 16–20 h. Clinical carbapenem breakpoints for susceptibility/resistance were ≤ 1/≥4 mg/L for imipenem and meropenem and ≤ 0.5/≥2 mg/L for ertapenem.

### Disk Diffusion Method

Disk diffusion tests for imipenem, meropenem, and ertapenem (Oxoid, Basingstoke, United Kingdom) were carried out according to CLSI M2 (18). The content of three antimicrobials in each disk was 10 μg. The disk was pasted to the MH agar plate using sterile tweezers and inoculated with 0.5 McFarland standard suspension. Incubated these plates at 35°C for 16–20 h, the diameter of the inhibition zone was measured with a vernier caliper. The zone diameter ≥23 mm indicated that the strain was susceptible to imipenem and meropenem and ≥22 mm to ertapenem, whereas the zone diameter ≤ 19 mm indicated that the strain was resistant to imipenem and meropenem and ≤ 18 mm to ertapenem.

### Screening of Carbapenemase Genes

Polymerase chain reaction (PCR) and Sanger sequencing were used to screen out carbapenemase genes, including KPC, NDM, VIM, IMP, and OXA-48. The oligonucleotide sequences of the primers were listed in Supplementary Table 2 (9, 19, 20). The QIAquick PCR Purification Kit was used to purify the PCR product, #REF is 28104. The PCR products were sequenced and analyzed using BLAST (http://www.ncbi.nlm.nih.gov/BLAST). Our BLAST cut-off is that the percent identity must be 100%.

### Data Analysis

Using BMD as a gold stand, the categorical agreement (CA), essential agreement (EA), minor error (MIE), major error (ME), and very major error (VME) were calculated (21, 22). Results were considered CA when isolates had the same susceptible, intermediate, susceptible-dose dependent, and resistant category with the BMD method category result. Results were considered EA when the MIC obtained with the BD Phoenix NMIC-413 panel was within one doubling dilution step (two-fold serial) of the MIC value established by the BMD method. Results were considered ME when the BMD method result was susceptible and the BD Phoenix NMIC-413 panel is resistant. Results were considered MIE when one result was intermediate and the other was susceptible or resistant. Results were considered VME when the BMD method result was resistant and the BD Phoenix NMIC-413 panel was susceptible. The calculation formulas of related parameters were shown in Supplementary Table 3. The Spearman correlation coefficients (*P*-value) were calculated by SPSS 26.0. The linear regression curve was performed using GraphPad Prism8, and the *R*^2^-value was obtained at same time.

## Results

### Isolates Information

**Figure 1:**
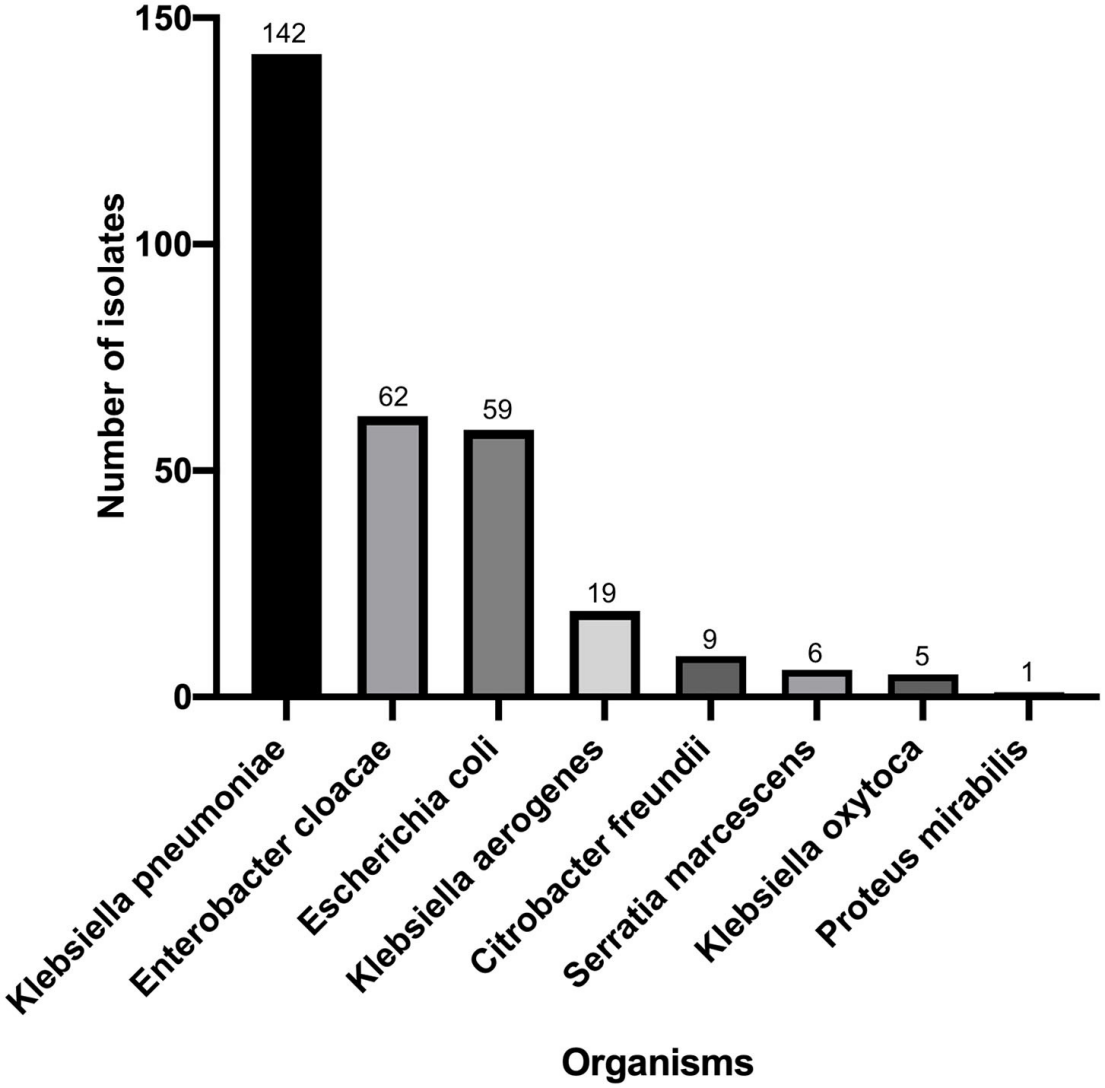
Distribution of *Enterobacterales* tested in the study (*n* = 303).

**Figure 2:**
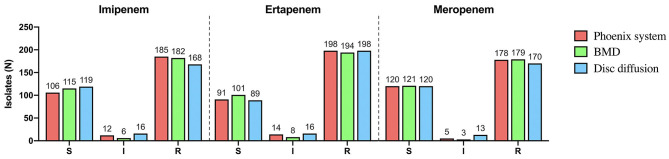
The susceptibility of 303 isolates to imipenem, ertapenem and meropenem with different methods.

*Klebsiella pneumoniae* (*n* = 142) accounted for the highest proportion, followed by *Enterbacter cloacae* (*n* = 62), *Enterbacter coli* (*n* = 59), *Klebsiella aerogenes* (*n* = 19), *Citrobacter freundii* (*n* = 9), *Serratia marcescens* (*n* = 6), *Klebsiella oxytoca* (*n* = 5), and *Proteus mirabilis* (*n* = 1) (Figure 1). There were 195 strains resistant to at least one of the 3 antimicrobials: imipenem, meropenem, and ertapenem. The susceptibility of 303 isolates to 3 carbapenems with different methods were shown in Figure 2. The numbers of imipenem, ertapenem, and meropenem resistant strains were 182, 194, and 179 by BMD, respectively. All three antibiotics were resistant to 177 strains, and at least one of them was resistant to 195 strains. There were 195 CRE and 108 carbapenem-susceptible *Enterobacterales* (CSE) in this study. The resistance rate of the BD Phoenix NMIC-413 (61.06, 65.35, and 58.75%) was in general agreement with BMD (60.07, 64.03, and 59.08%), and better than disk diffusion (55.45, 65.35, and 56,12%) (*P* > 0.05).

### Genotype Determination

The genotypes determined by PCR and Sanger sequencing demonstrated different resistance mechanisms which were shown in Table 1. The highest proportion was the KPC-2 carbapenemase gene (100/195), followed by IMP (33/195), NDM (28/195), VIM (7/195), and OXA-48(2/195). Two isolates produced both IMP-1 and VIM-1. A total of 27 CRE isolates were carbapenemase gene negative in this study. KPC-2 was the most prevalent carbapenemase gene in *K. pneumoniae* strains, while IMP and NDM accounted for the majority gene type in *E. cloacae* and *C. freundii*. All carbapenem-susceptible isolates (*N* = 108) were proved no carbapenemase genes by PCR method.

**Table 1:** The enzyme type and strain types of 195 CRE strains.

**Organism**	**Number and percentage of isolates with different resistance mechanisms [*****n*** **(%)]**					
	**KPC**	**IMP**	**NDM**	**VIM**	**OXA-48**	**Carbapenemase negative**
*Klebsiella pneumoniae* (110)	84 (76.36)	10 (9.09)	6 (5.45)	3 (2.73)	0	7 (6.36)
*Enterobacter cloacae* (38)	4 (10.53)	11 (28.95)	14 (36.84)	0	0	9 (23.68)
*Escherichia coli* (21)	6 (28.57)	2 (9.53)	6 (28.57)	1 (4.76)	2 (9.52)	4 (19.05)
*Klebsiella aerogenes* (8)	3 (37.50)	1 (12.50)	0	0	0	4 (50.00)
*Citrobacter freundii* (7)	0	6 (85.71)	1 (14.29)	0	0	0
*Serratia marcescrns* (5)	1 (20.00)	0	0	1 (20.00)	0	3 (60.00)
*Klebsiella oxytoca* (5)	1 (20.00)	3 (60.00)	1(20.00)	0	0	0
*Proteus mirabilis* (1)	1 (100.00)	0	0	0	0	0

*KPC, Klebsiella pneumoniae carbapenemase; NDM, New Delhi metallo-beta-lactamase; VIM, Verona integron-borne metallo-beta-lactamase; IMP, imipenemase; OXA, oxacillinase*.

### Performance of BD NMIC-413 Panel vs. Disk Diffusion Method

**Figure 3:**
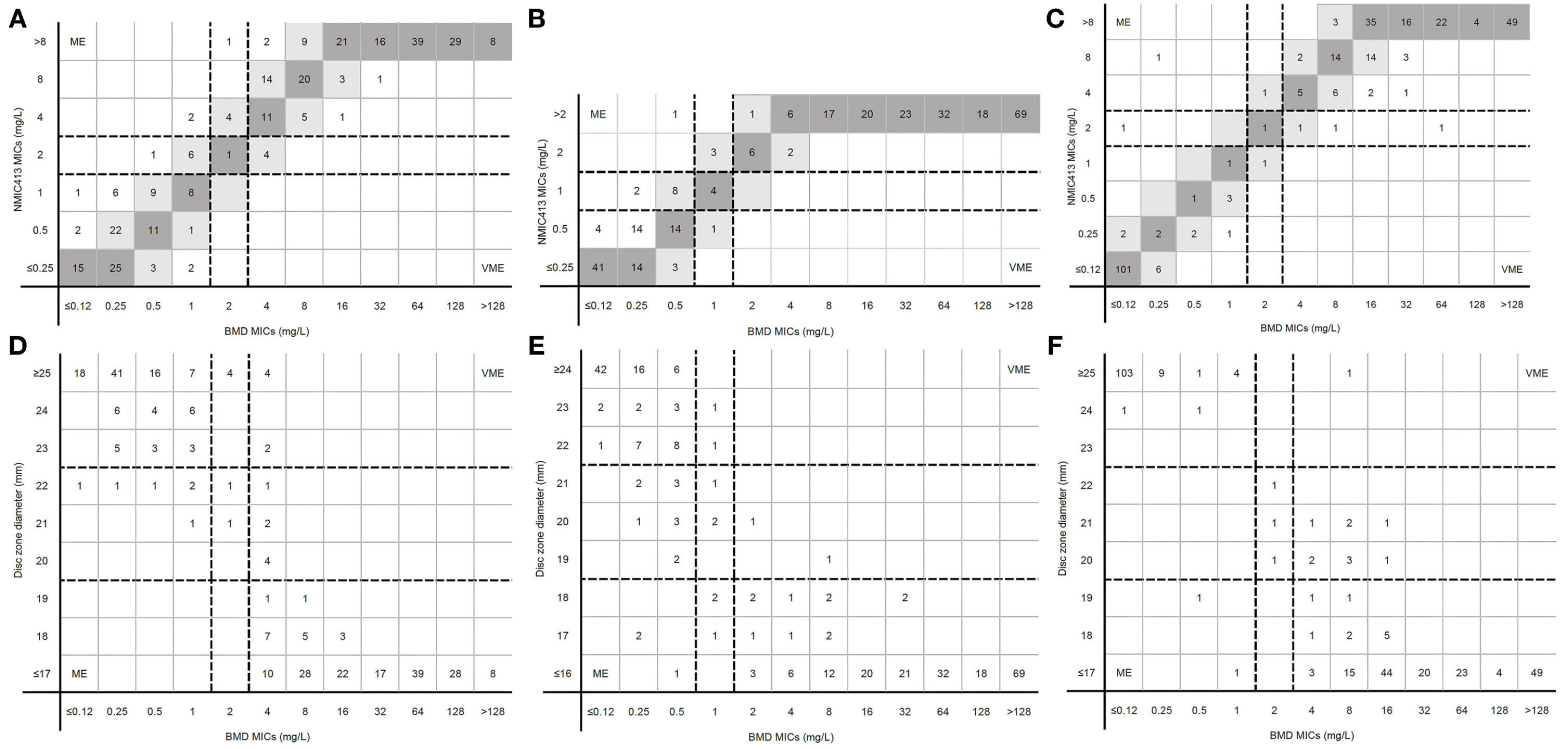
MICs determined by BD Phoenix NMIC-413 panel, disk diffusion and broth microdilution. **(A)** (imipenem), **(B)** (ertpenem), and **(C)** (meropenem) were the results of BD Phoenix NMIC-413 vs. broth microdilution; **(D)** (imipenem), **(E)** (ertpenem), and **(F)** (meropenem) were the results of disk diffusion vs. broth microdilution. Dark gray expresses identical MIC, and light gray indicates 2-fold difference between the BMD and NMIC-413 panel MICs. Dotted lines show the clinical breakpoints for each antibiotics.

Figure 3 and Table 2 showed CAs, EAs, and the number of MIE, ME, and VME for 303 clinical *Enterobacterales* isolates. CA and EA were all above 90%, and the CA of the three antibiotics in the BD Phoenix NMIC-413 was greater than the disk diffusion.

**Table 2:** Performance of the BD Phoenix NMIC-413 and disk diffusion compared with BMD for *Enterobacterales* isolates.

**Antimicrobial agent**	**BD Phoenix NMIC-413 vs. BMD** ***N*** **(%)**					**Disk diffusion vs. BMD** ***N*** **(%)**			
	**CA**	**EA**	**MIE**	**ME**	**VME**	**CA**	**MIE**	**ME**	**VME**
Imipenem	284 (93.73)	284 (93.73)	16 (5.28)	2 (1.75)	0	283 (93.40)	14 (4.62)	0	6 (3.28)
Ertapenem	288 (95.05)	296 (97.69)	14 (4.62)	1 (0.99)	0	285 (94.06)	15 (4.95)	3 (2.97)	0
Meropenem	296 (97.69)	292 (96.37)	6 (1.98)	1 (0.83)	0	289 (95.38)	10 (3.30)	2 (1.65)	1(0.56)

*S, susceptible; I, intermediate; R, resistant; BMD, broth microdilution; CA, categorical agreement; EA, essential agreement; MIE, minor error; ME, major error; VME, very major error*.

For imipenem, CA and MIE of the BD Phoenix NMIC-413 were similar with the disk diffusion. However, the VME of BD Phoenix NMIC-413 was 0%, while the disk diffusion was 3.28%. The ME rate of disk diffusion was close to 3%, while the BD Phoenix NMIC-413 was only 0.99%. For meropenem, the CA, MIE, ME, VME of BD NMIC-413 and disk diffusion was 97.69 vs. 95.38%, 1.98 vs. 3.30%, 0.83 vs. 1.65%, 0 vs. 0.56%. All the indexes of BD Phoenix NMIC-413 were better than disk diffusion. The performance of ertapenem of both methods is comparable, the CA, MIE, ME, VME was 95.05 vs. 94.06%, 4.62 vs. 4.95%, 0.99 vs. 2.97%, 0 vs. 0%.

**Figure 4:**
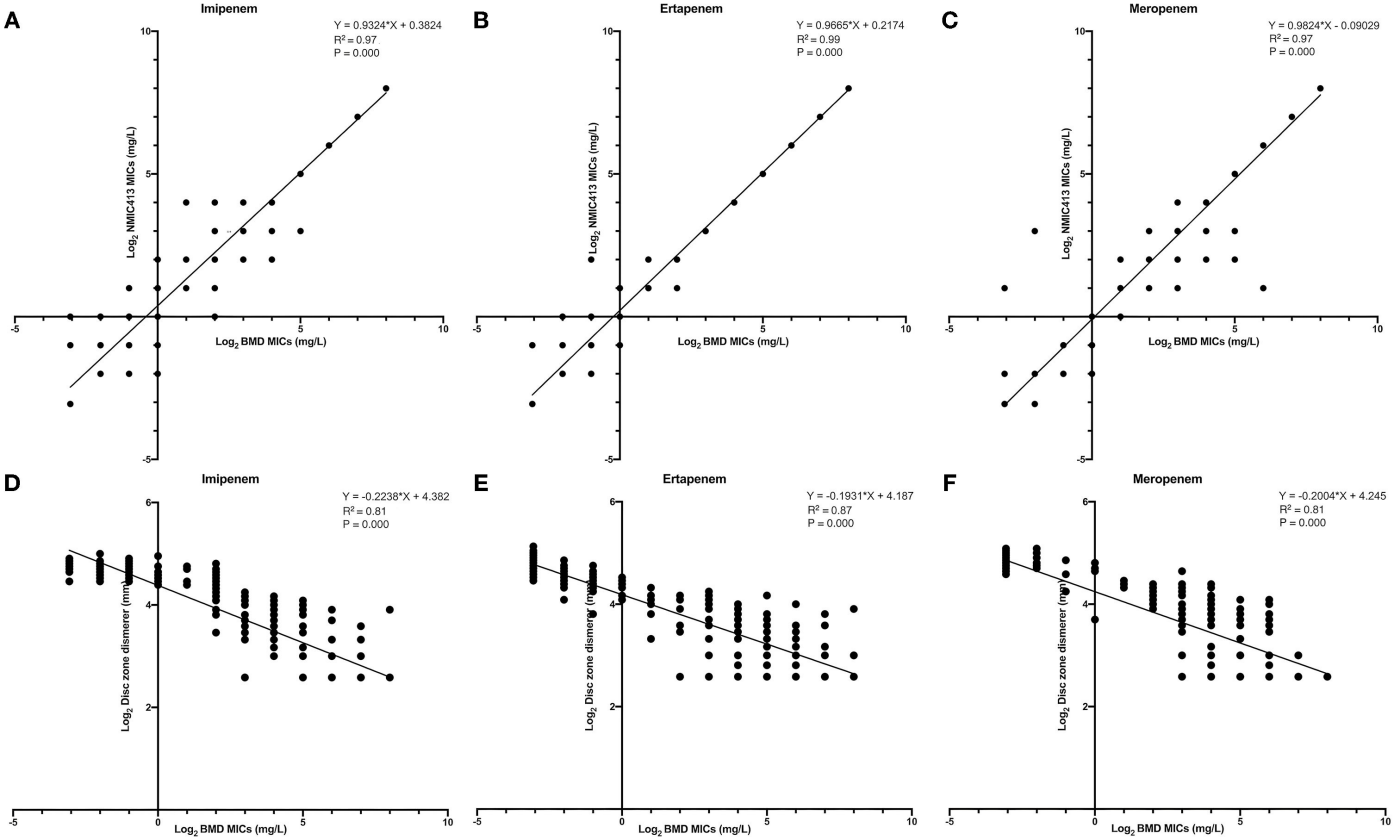
The linear regression curve between the MICs determined by BD NMIC-413, disk diffusion and broth microdilution. **(A)** (imipenem), **(B)** (ertpenem), and **(C)** (meropenem) were curves between BD NMIC-413 and broth microdilution; **(D)** (imipenem), **(E)** (ertpenem), and **(F)** (meropenem) were curves between disk diffusion and broth microdilution.

Figures 4A–C showed the linear regression curve between the MICs determined by BD NMIC-413 and BMD. The *R*^2^-value of imipenem, ertapenem, and meropenem was 0.97, 0.99, and 0.97, respectively. The *P*-values were all <0.001. Therefore, the BD NMIC-413 had the best performance in the detection of ertapenem. Figures 4D–F showed the linear regression curve between disk diffusion and BMD. The *R*^2^-values were 0.81–0.87, of which the *R*^2^-value of ertapenem was the largest (0.87).

Figures 3A–C displayed the MICs distribution of imipenem, ertapenem, and meropenem by BMD and the BD Phoenix NMIC-413. Most MEs were clustered near the susceptibility breakpoint. For imipenem and ertapenem, the resistance rates detected by BD Phoenix NMIC-413 were higher than BMD.

Figures 3D–F displayed the MICs and disk zoom diameter of three antibiotics by BMD and disk diffusion. Only in ertapenem, the number of drug resistance measured by the disk diffusion method was more than that of BMD (198 vs. 194), and the number of drug resistance of the other two drugs were nearly 10 less (imipenem: 168 vs. 182; meropenem: 170 vs. 179).

### Performance Evaluation Against CRE Strains With Different Genotypes

For imipenem, the BD NMIC-413 and disk diffusion method showed a CA rate of 62.96 and 74.07% separately against carbapenemase-non-producing CREs. The disk diffusion performed not well in IMP-producing CREs, with CA of 72.73% and VME of 9.09%. There was a KPC-producing *K. pneumoniae*, an OXA-48-producing *Es. coli*, and an IMP-producing *K. aerogenes* in the BD NMIC-413 that belong to MIE, the MIC detected by BMD and BD NMIC-413 were 4 and 2 mg/L, respectively.

For ertapenem, two methods performed well in the detection of different enzyme types, only disk diffusion had a slightly lower CA (93.94%) in the detection of IMP type isolates. Ertapenem was the best to detect CRE among the three antibiotics.

For meropenem, the EA of IMP-producing CREs was <90% (87.88%) when detected by the BD Phoenix NMIC-413. When testing the IMP/VIM producing isolates or carbapenemase-non-producing isolates, the disk diffusion method showed that the CA was <90%, while the VME of IMP-producing isolates was >3% (3.13%). There was one KPC-producing *Es. coli*, one carbapenemase-non-producing *E. cloacae*, and one carbapenemase-non-producing *K. pneumoniae* in BD NMIC-413 belong to MIE. These results of BMD were resistant (MIC was 8, 64, and 4 mg/L, respectively), while BD NMIC-413 were intermediate (MIC all was 2 mg/L). Meanwhile, there was also one IMP-producing *K. pneumoniae* and one OXA-48-producing *Es. coli*, the results detected by BMD were intermediate (MIC for both of them was 2 mg/L), while BD NMIC-413 were resistant and susceptible (MIC was 4 and 1 mg/L). Another one KPC-producing *K. pneumoniae* was also MIE, and the result of BMD was susceptible (MIC was ≤ 0.12 mg/L) while NMIC413 results were intermediate (MIC was 2 mg/L).

Table 3 showed the rates of CAs, EAs, and the number of MIE and VME for 195 resistant isolates with the three antibiotics.

**Table 3:** Performance of the BD Phoenix NMIC-413 and disk diffusion compared with BMD for 195 CREs.

**Enzyme**	**BD Phoenix NMIC-413 vs. BMD** ***N*** **(%)**				**Disk diffusion vs. BMD** ***N*** **(%)**		
	**CA**	**EA**	**MIE**	**VME**	**CA**	**MIE**	**VME**
**Imipenem**							
KPC (100)	99 (99.00)	99 (99.00)	1 (1.00)	0	99 (99.00)	1 (1.00)	0
NDM (28)	28 (100)	28 (100)	0	0	28 (100)	0	0
IMP (33)	32 (96.97)	33 (100)	1 (3.03)	0	24 (72.73)	6 (18.18)	3 (9.09)
VIM (7)	7 (100)	7 (100)	0	0	7 (100)	0	0
OXA-48 (2)	1 (50.00)	2 (100)	1(50.00)	0	2 (100)	0	0
no enzyme (27)	17 (62.96)	26 (96.30)	10 (37.04)	0	20 (74.07)	5 (18.52)	3 (11.11)
**Ertapenem**							
KPC (100)	99 (99.00)	99 (99.00)	0	0	99 (99.00)	0	0
NDM (28)	28 (100)	28 (100)	0	0	28 (100)	0	0
IMP (33)	33 (100)	33 (100)	0	0	31 (93.94)	2 (6.06)	0
VIM (7)	7 (100)	7 (100)	0	0	7 (100)	0	0
OXA-48 (2)	2 (100)	2 (100)	0	0	2 (100)	0	0
No enzyme (27)	27 (100)	27 (100)	0	0	27 (100)	0	0
**Meropenem**							
KPC (100)	99 (99.00)	96 (96.00)	1 (1.00)	0	98 (98.00)	1 (1.00)	0
NDM (28)	28 (100)	28 (100)	0	0	28 (100)	0	0
IMP (33)	31 (93.94)	29 (87.88)	2 (6.06)	0	27 (81.82)	5 (15.15)	1 (3.13)
VIM (7)	7 (100)	7 (100)	0	0	6 (85.71)	1 (14.29)	0
OXA-48 (2)	1 (50.00)	2 (100)	1 (50.00)	0	2 (100)	0	0
No enzyme (27)	25 (92.59)	25 (92.59)	2 (7.41)	0	23 (85.19)	3 (11.11)	0

*S, susceptible; I, intermediate; R, resistant; BMD, broth microdilution; CA, categorical agreement; EA, essential agreement; MIE, minor error; VME, very major error; KPC, Klebsiella pneumoniae carbapenemase; NDM, New Delhi metallo-beta-lactamase; VIM, Verona integron-borne metallo-beta-lactamase; IMP, imipenemase; OXA, oxacillinase*.

## Discussion

In prior studies, the BD Phoenix NMIC-500 and NMIC-203 panels have been reported for the performance evaluation of negative bacilli, and PMIC-84 panel has reported for positive cocci (15, 23), including identification and antimicrobial susceptibility testing evaluation (24). In this study, the performance of the BD Phoenix NMIC-413 system used for CRE testing was compared with the traditional disk diffusion method, while BMD was used as the reference method. Compared with BMD and disk diffusion, the BD Phoenix NMIC-413 panel had higher CA/EA and lower ME/MIE/VME. For carbapenemase genes negative CRE isolates, the CA rates of imipenem were <75%, while the other two antibiotics were all >90%. Imipenem was often degraded by the enzyme dehydropeptidase-1 (DHP-1), hence the co-administration with a DHP-1 inhibitor such as cilastatin was required (25). Later meropenem and ertapenem demonstrated increased stability to DHP-1 and were administered without a DHP-1 inhibitor (25). So meropenem and ertapenem are more stable than Imipenem. For intermediate isolates, the MICs obtained by BD Phoenix system NMIC-413 panel were probably higher than BMD. Considering that the BD Phoenix system is a broth-based microdilution method that not only measures turbidity but also detects the bacterial growth utilizing the redox indicator (26), enabling it to detect resistant bacteria with high sensitivity. In addition, the BD Phoenix system had fewer manual operations, which was simpler and more convenient compared with the BMD method. The advantages and disadvantages of the three methods were shown in Supplementary Table 4.

Haffler et al. (27) have evaluated one panel of the BD Phoenix system and showed that the CA of ertapenem and meropenem were 94 and 50%. Hogan et al. (28) have evaluated the AST of VITEK 2 (bioMérieux, France) with Gram-negative bacteria by blood culture and reported that the CA and EA were 86.5 and 84.6, 96.2, and 96.2% for meropenem and ertapenem, respectively. In our study, the CA and EA of the BD Phoenix system NMIC-413 panel were ranged from 93 to 98%.

However, the BD Phoenix system NMIC-413 panel can only obtain MICs of the designated antibiotics, so it cannot be used for taxonomic identification of the selected isolates. However, the BD Phoenix CPO panel can detect the MIC and classification of CRE at the same time. It can divide CRE into three categories: A (KPC), B (NDM, IMP, and VIM), and D (OXA-48). Saad Albichr et al. (29) evaluated the performance of the automated BD Phoenix CPO Detect-test for detection and Ambler classification of carbapenemases in *Enterobacterales, P. aeruginosa*, and *A. baumannii* complex, the overall sensitivity and specificity were 89.7 and 83.5%, respectively, 68.9 and 62.1% for *P. aeruginosa*, respectively. Although the BD Phoenix system can detect the classification of CRE expediently, the accuracy needed to improve, it also needs other methods to verify.

KPC type enzyme is the most prevalent carbapenemase in China (30). A. Antonelli has investigated the sensitivity of 6 different commercial methods (Sensititre, Microscan, Vitek2, Etest, Kirby-Bauer, and MIC strip) on KPC-producing isolates (31). In that study, the CA rates of imipenem, meropenem, and ertapenem were 16.7–51.8%, 14.8–79.6%, and 83.3–96.3%, respectively, which were much lower than NMIC-413 panel evaluated in this study.

The BD phoenix NMIC-413 panel also had a notable limitation in this study. There are not enough *bla*_VIM_ and *bla*_OXA_ producing strains among the isolates tested in this study. Mainly because these two carbapenemase types are very rare in China (32, 33). From 2010 to 2019, we isolated only 7 *bla*_VIM_ and 2 *bla*_OXA_ producing strains in our laboratory. Now we do not have enough VIM and OXA-48 to make up for this limitation. In the next work, we will deliberately save these two types of strains to facilitate future research.

## Conclusion

In conclusion, for the BD Phoenix NMIC-413 panel, CA and EA rates > 93%, MIE rates <5%, ME rates <1.75%, and VME rates were 0%, across the three drugs. It showed acceptable performance as alternatives to the BMD method for clinical treatment explanation.

## Data Availability Statement

The original contributions presented in the study are included in the article/Supplementary Material, further inquiries can be directed to the corresponding authors.

## Ethics Statement

The protocol has been reviewed by the Human Research Ethics Committee of the Institutional Review Board (IRB) of the Peking Union Medical College Hospital and since all bacterial strains were from residual samples used in clinical diagnosis or strains from their subcultures, it met the criteria for exemption. This project did not involve any patient information nor did it affect the normal diagnosis and treatment of patients, and after consultation with the IRB, formal ethical approval was reviewed and waived; written patient consent was not required (Ethics Approval Number: S-K677).

## Author Contributions

QY and YX conceived and designed the experiments. JZ, GZ, PJ, and YZ performed the experiments, analyzed the data, and wrote the paper. All authors approved the final version of the manuscript.

## Conflict of Interest

The authors declare that the research was conducted in the absence of any commercial or financial relationships that could be construed as a potential conflict of interest.
