# Supplementary material for: Performance Evaluation of BD Phoenix NMIC-413 Antimicrobial Susceptibility Testing Panel for Imipenem, Meropenem, and Ertapenem Against Clinical Carbapenem-Resistant and Carbapenem-Susceptible Enterobacterales
Source: Front Med (Lausanne). 2021 Apr 14;8:643194. doi: 10.3389/fmed.2021.643194 (PMC8079628; doi:10.3389/fmed.2021.643194)
Supplement: Supplementary file 2 [file Table_2.docx]

**Supplement Table2.** Oligonucleotide sequences of the primers used in this study.

| Target gene | Primer name | sequence | Product size |
| --- | --- | --- | --- |
| *bal_KPC_* | KPC-F | 5'-TGTCACTGTATCGCCGTC-3' | 1010 |
|  | KPC-R | 5'-CTCAGTGCTCTACAGAAAACC-3' |  |
| *bal_IMP_* | IMP1-F | 5'-TGAGCAAGTTATCTGTATTC-3' | 740 |
|  | IMP1-R | 5'-TTAGTTGCTTGGTTTTGATG-3' |  |
| *bal_VIM_* | VIM1-F | 5'-TTATGGAGCAGCAACCGATGT-3’ | 920 |
|  | VIM1-R | 5'-CAAAAGTCCCGCTCCAACGA-3’ |  |
| *bal_NDM_* | NDM-F | 5'-TCGCCCCATATTTTTGCTACAG-3’ | 1012 |
|  | NDM-R | 5'-CGATCCTTCCAACTCGTCGC-3’ |  |
| *bal_OXA-48_* | OXA-F  OXA-R | 5’-TTGGTGGCATCGATTATCGG-3’  5’-GAGCACTTCTTTTGTGATGGC-3’ | 744 |

**Abbreviations:** KPC, Klebsiella pneumoniae carbapenemase; NDM, New Delhi metallo-beta-lactamase; VIM, Verona integron-borne metallo-beta-lactamase; IMP, imipenemase; OXA, oxacillinase; F, Forward; R, Reverse.
