# Supplementary material for: Performance Evaluation of BD Phoenix NMIC-413 Antimicrobial Susceptibility Testing Panel for Imipenem, Meropenem, and Ertapenem Against Clinical Carbapenem-Resistant and Carbapenem-Susceptible Enterobacterales
Source: Front Med (Lausanne). 2021 Apr 14;8:643194. doi: 10.3389/fmed.2021.643194 (PMC8079628; doi:10.3389/fmed.2021.643194)
Supplement: Supplementary file 3 [file Table_3.docx]

**Supplement Table3.** Description of related parameters.

| parameters | Description |
| --- | --- |
| category agreement (CA) | $\% CA= \frac{N_{CA}\cdot100}{N}$; *N*_CA_ is the number of microbial isolates with the same susceptible, intermediate, susceptible-dose dependent, and resistant category as the reference or comparator method category result; *N* is the total number of microbial isolates tested |
| essential agreement (EA) | MIC result obtained with the antimicrobial susceptibility testing system that is within one doubling dilution step (two-fold serial) for bacteria from the MIC value established with the reference method; $\% EA= \frac{N_{EA}\cdot100}{N}$; *N*_EA_ is the number of microbial isolates with an EA; *N* is the total number of microbial isolates tested |
| major error (ME) | $\% ME=\frac{N_{ME}\cdot100}{total isolates susceptible by reference method}$ |
| minor error (MIE) | $\% MIE= \frac{N_{MIE}\cdot100}{N_{T}}$; *N*T is the total number of isolates tested |
| very major error (VME) | $\% VME=\frac{N_{VME}\cdot100}{total isolates resistant by reference method}$ |
