# Supplementary material for: Performance Evaluation of BD Phoenix NMIC-413 Antimicrobial Susceptibility Testing Panel for Imipenem, Meropenem, and Ertapenem Against Clinical Carbapenem-Resistant and Carbapenem-Susceptible Enterobacterales
Source: Front Med (Lausanne). 2021 Apr 14;8:643194. doi: 10.3389/fmed.2021.643194 (PMC8079628; doi:10.3389/fmed.2021.643194)
Supplement: Supplementary file 4 [file Table_4.docx]

**Supplement Table4.** The advantage and disadvantage of three methods.

| Method | Reagents and supplies | Description | Advantages | Disadvantages |
| --- | --- | --- | --- | --- |
| BMD | antibiotic susceptibility test plate (need to be prepared manually);  MH broth (need to be prepared manually); sterile water; spectrophotometric device | AST plates and MH broths need to be prepared manually; antibiotic powder dosing need to be weighed manually; AST plates need to be kept in -80℃refrigerator; The process of transferring the MH broth of the inoculated bacteria to the AST plate is done manually | high accuracy | too many manual operations |
| BD Phoenix NMIC-413 | BD identification broth; BD Antibiotic susceptibility test broth; BD Antibiotic susceptibility test redox indicator; BD Phoenix NMIC-413 panel; spectrophotometric device | The reagents and supplies required are purchased from BD company, less manual work is required | high accuracy simple instruction | more reagents are required |
| Disc diffusion | sterile water; spectrophotometric device; MH agar plate; antibiotic susceptibility test disc | The required reagents and supplies can be  purchased without the need for manual preparation | simple instruction | poor accuracy |
